# Genomic and Immune Correlates of EZH2 Expression and Activity in Olfactory Neuroblastoma

**DOI:** 10.1002/hed.70076

**Published:** 2025-11-12

**Authors:** Elisabetta Xue, Tolulope Adeyelu, Harris Krause, Andrew Elliott, Ranee Mehra, Heloisa Soares, Emil Lou, Ari Vanderwalde, David Spetzler, Dara Bracken‐Clarke, Nyall R. London, James L. Gulley, Charalampos S. Floudas

**Affiliations:** ^1^ Center for Immuno‐Oncology CCR, NCI, NIH Bethesda Maryland USA; ^2^ CARIS Life Sciences Phoenix Arizona USA; ^3^ University of Maryland Marlene and Stewart Greenebaum Cancer Center, University of Maryland School of Medicine Baltimore Maryland USA; ^4^ Huntsman Cancer Institute, University of Utah Salt Lake City Utah USA; ^5^ Masonic Cancer Center, University of Minnesota Minneapolis Minnesota USA; ^6^ Department of Otolaryngology‐Head and Neck Surgery Johns Hopkins University School of Medicine Baltimore Maryland USA; ^7^ Sinonasal and Skull Base Tumor Section, Surgical Oncology Program, CCR, NCI, NIH Bethesda Maryland USA

## Abstract

**Purpose:**

Olfactory neuroblastoma (ONB) is a rare sinonasal malignancy with limited therapeutic options in the recurrent/metastatic setting; little is known regarding its responsiveness to immunotherapy. Inhibition of enhancer of zeste homolog 2 (EZH2) has been shown to improve T‐cell‐mediated killing and susceptibility to immune checkpoint inhibitors in a variety of cancers. We aimed to evaluate the expression and activity of EZH2 in ONB and its association with immune characteristics.

**Materials and Methods:**

We studied a cohort of 36 ONB real‐world patient samples that underwent molecular profiling at a centralized lab (Caris Life Science). To infer EZH2 methyltransferase activity, we adopted an EZH2 gene repression signature (ERS) score: ONB samples were stratified into ERS‐low and ERS‐high subgroups, corresponding to high and low inferred EZH2 methyltransferase activity, respectively. Transcriptomic data were utilized to calculate the T‐cell‐inflamed (TCI) score and mitogen‐activated protein kinase (MAPK) pathway activation score (MPAS). Tumor immune microenvironment composition was inferred from tumor‐derived bulk RNA sequencing data. We analyzed immunologic differences between ERS‐low and ERS‐high ONB.

**Results:**

In ERS‐high ONB, we observed a higher expression of immune‐related genes, a higher proportion of TCI tumors, and an enrichment in inflammatory pathways. ERS‐high ONB also displayed increased macrophages, and to a lesser extent, B cells and CD8^+^ T cell infiltration in the tumor microenvironment. Also, ERS‐high was associated with increased MPAS, potentially identifying ONB with increased susceptibility to MAPK inhibitors. These data were confirmed in an independent validation cohort using a publicly available dataset.

**Conclusions:**

Taken together, our data suggest that low EZH2 activity is associated with a more immunogenic microenvironment, paving the way for potential combinations of EZH2 inhibitors with checkpoint blockade in ONB.

## 1 Introduction

Olfactory neuroblastoma (ONB) is a rare sinonasal cancer with neuroendocrine features and similarity to globose basal cells, neuronal progenitors present in the olfactory neurosensory epithelium [1]. It has few systemic therapeutic options in the recurrent and metastatic setting and is generally characterized by low tumor mutational burden (TMB) [2] with limited evidence for the activity of immune checkpoint inhibitors [3].

The enhancer of zeste homolog 2 (EZH2) is a core protein of the epigenetic modifier methyltransferase polycomb repressive complex 2 (PRC2), which catalyzes histone H3 methylation on lysine 27 (H3K27me3) and regulates cell cycle progression, promoting self‐renewal and hindering cell differentiation [4, 5]. Gain‐of‐function mutations and overexpression of *EZH2* have been associated with tumor invasion, chemotherapy resistance, and poor prognosis in different tumor types [6, 7]. Both enzymatic (canonical) and other nonenzymatic (noncanonical) EZH2 activities are antagonistically regulated by the Switch/Sucrose Non‐fermentable (SWI/SNF) chromatin remodeling complex [8], of which *SMARCB1* (INI1) is a major component. In *SMARCB1*‐deficient cancers, such as epithelioid sarcoma (ES), loss of *SMARCB1*‐mediated antagonistic activity leads to unopposed activity of EZH2/PRC2 and drives tumor progression [9]. This activity has been explored and leveraged therapeutically with tazemetostat, the only currently approved treatment for *SMARCB1*‐deficient ES that inhibits EZH2 methyltransferase activity [10]. Other components of the SWI/SNF complex, like *SMARCA4* and *ARID1A*, are being increasingly studied, shedding light on the complex interaction between EZH2/PRC2 and SWI/SNF complexes [11].

Overexpression of EZH2 has been associated with resistance to immunotherapy. In hepatocellular carcinoma, EZH2‐mediated downregulation of programmed death‐ligand 1 (PD‐L1) expression was reported through the increase of H3K27me3 levels of PD‐L1 (*CD274*) and transcription factor *IRF1* [12]. EZH2‐mediated downregulation of β2‐microglobulin, HLA Class I molecules, and IFNγ signaling genes might also favor a nonimmunogenic environment [13, 14]. Inhibition of EZH2 in leukemia relapsing after allogeneic hematopoietic cell transplant was sufficient to rescue HLA Class II expression and, indirectly, donor T‐cell‐mediated tumor killing [15]. In melanoma models, exposure to checkpoint blockade resulted in increased EZH2 expression and activity, which in turn favored T cell exhaustion and reduced tumor immunogenicity, leading to treatment resistance. In this model, EZH2 inhibition was sufficient to restore immunotherapy sensitivity and was followed by increased tumor‐infiltrating lymphocytes [16]. Similar findings have been reported in prostate and head and neck cancers [13, 17].

In normal murine olfactory epithelium, EZH2 expression has been shown to be confined to the proliferative globose basal cells compartment, and its inhibition resulted in decreased cell proliferation [18]. In ONB, Finlay et al. documented higher EZH2 expression in high‐grade tumors, specifically in the proliferating compartment, confirmed by positive co‐staining of Ki‐67 and EZH2 by immunohistochemistry (IHC) [1, 19]. Herein, we aimed to assess the genomic and transcriptomic profiles associated with an EZH2 gene repression signature (ERS) score to infer EZH2 methyltransferase activity and its correlation with immune‐related features in a cohort of real‐world ONB patient samples.

## 2 Materials and Methods

### 2.1 Specimens

We retrospectively examined the molecular profile of 36 ONB tumor specimens from either primary (*N* = 18) or metastatic (*N* = 18) site; the median age at diagnosis was 59 years (range = 27–79), and 17 (47.2%) were male; these specimens underwent NextGen Sequencing of DNA (592‐gene panel or whole‐exome sequencing [WES]) and whole‐transcriptome sequencing (WTS) in a CLIA certified clinical laboratory (Caris Life Sciences, Phoenix, AZ, USA). Given that ES are virtually always characterized by a *SMARCB1*/INI1 loss or inactivation, leading to unopposed activity of the EZH2/PRC2 complex, we used a cohort of 78 ES specimens for comparison with our ONB study population. The study follows guidelines provided by the Declaration of Helsinki, Belmont Report, and U.S. Common Rule. In accordance with compliance policy 45 CFR 46.101(b), this study was conducted using retrospective, de‐identified clinical data; patient consent was not required, and the study was considered IRB exempt.

### 2.2 DNA Next‐Generation Sequencing

A targeted 592‐gene panel or WES was performed using genomic DNA isolated from microdissected formalin‐fixed, paraffin‐embedded tumor samples. The 592‐gene panel was sequenced using the NextSeq platform (Illumina Inc., San Diego, CA, USA). A custom‐designed SureSelect XT assay was used to enrich 592 whole‐gene targets (Agilent Technologies, Santa Clara, CA, USA). WES was performed using the Illumina NovaSeq 6000 sequencer (Illumina Inc.). A hybrid pull‐down panel of baits designed to enrich for 700 clinically relevant genes at high coverage and high read depth was used, along with another panel designed to enrich for an additional > 20 000 genes at lower depth. 592‐gene and WES assays were cross‐validated and showed highly concordant results. Matched normal tissue was not sequenced.

### 2.3 Identification of Genetic Variants

Genetic variants identified were interpreted by board‐certified molecular geneticists and categorized as “pathogenic,” “likely pathogenic,” “variant of unknown significance,” “likely benign,” or “benign,” according to the American College of Medical Genetics and Genomics (ACMG) standards. When assessing mutation frequencies of individual genes, “pathogenic” and “likely pathogenic” were counted as mutations, while “benign,” “likely benign” variants, and “variants of unknown significance” were excluded.

### 2.4 RNA WTS

Formalin‐fixed paraffin‐embedded (FFPE) specimens underwent pathology review to determine percentage tumor content and tumor size; a minimum of 10% of tumor content in the area for microdissection was required. Qiagen RNA FFPE kit (Qiagen, Germantown, MD, USA) was used, and the RNA quality and quantity were determined using the Agilent TapeStation (Agilent Technologies). Biotinylated RNA baits were hybridized to the synthesized and purified cDNA targets, and the bait‐target complexes were amplified in a post‐capture PCR reaction. The resultant libraries were quantified, normalized, and the pooled libraries were denatured, diluted, and sequenced. For transcript counting, transcripts per million molecules (TPM) were generated using the Salmon expression pipeline. Expression level was transformed and reported as log2TPM + 1 except as otherwise specified. We labeled ONB samples as neural or basal according to Classe et al. [20] using Ward's clustering method.

### 2.5 Immunotherapy‐Related Biomarkers, Signatures, and Immune Cell Infiltration

For both the 592‐gene and WES panels, TMB was measured by counting all nonsynonymous missense, nonsense, in‐frame insertion/deletion (indels), and frameshift mutations (fs) found per tumor that had not been previously described as germline alterations in dbSNP151, Genome Aggregation Database (gnomAD) databases, or benign variants identified by Caris geneticists, with high‐TMB (TMB‐H) defined as ≥ 10 mutations per MegaBase. Microsatellite instability‐high (MSI‐H)/deficient DNA mismatch repair (dMMR) status was determined using NGS (for tumors tested with the NextSeq platform, 7000 target microsatellite loci were examined and compared to the reference genome hg19 from the University of California, Santa Cruz) and IHC (for MLH1 [M1 antibody], MSH2 [G2191129 antibody], MSH6 [44 antibody], and PMS2 [EPR3947 antibody, Ventana Medical Systems Inc., Tucson, AZ, USA]), respectively: these two methods generated highly concordant results; in the rare cases of discordant results, the status was determined by IHC. Expression of PD‐L1 was assessed by IHC through the 22C3 antibody (positive: tumor proportion score [TPS] ≥ 1%) or SP142 antibody (positive: ≥ 2+, ≥ 5%). All IHC was performed on FFPE sections of glass slides. Slides were stained using automated staining techniques, per the manufacturer's instructions, and were optimized and validated per CLIA/CAP and ISO requirements. They were all evaluated by a board‐certified pathologist.

Transcriptomic data were utilized to calculate T‐cell‐inflamed (TCI) score [21] and mitogen‐activated protein kinase (MAPK) pathway activation score (MPAS; [22]), two gene expression signatures predictive of response to checkpoint inhibitors and to tyrosine kinase inhibitors, respectively. The TCI score was calculated using 160 gene expression signatures, and TCI score < −80 is defined as non‐T‐cell inflamed, ≥ +80 as T‐cell inflamed, and the rest being intermediate. QuanTIseq, a computational deconvolution method that uses RNA transcripts that are known to be expressed in specific immune cell types, was used to estimate the immune cell type fractions from tumor‐derived bulk RNA sequencing data [23].

As a surrogate for EZH2 activity, we used a transcriptional “EZH2 Repression Signature” score (ERS; arbitrary units [AU], using 29 genes) previously developed in prostate cancer [17] derived by transcriptional analysis before and after EZH2 inhibition: high ERS is suggestive of higher transcriptional activity and thus lower EZH2 activity, and vice versa (Table S1). Three additional ERS variants were also investigated: equally weighted, immune regulatory genes excluded, and combined equally weighted with immune regulatory genes excluded. For the equally weighted, all genes were assigned a uniform weight of 1. In the immune regulatory gene exclusion scoring, 17 immune regulatory genes were removed from the calculation (see Supporting Information S1). An additional polycomb repression signature (PRS) previously developed in a prostate cancer model [24] was used for comparison.

To study transcriptional differences between ONB with high and low ERS, stratified by the median ERS of the ONB cohort, differential expression analysis was performed using PyDeseq2 [25]. Gene set enrichment analysis (GSEA) was performed on the WTS data using the Hallmark gene set collection from the Human Molecular Signature Database [26, 27].

### 2.6 Validation Dataset

The bulk RNA‐Seq data of 19 ONB samples described by Classe et al. [20] were downloaded from Gene Expression Omnibus (accession number GSE118995) and analyzed with DESeq2 [28]. The authors also reported genomic information on a larger sample size of 27 ONBs.

### 2.7 Statistical Analysis

Statistical analyses included Mann–Whitney *U*, Fisher's exact/chi‐square tests with Benjamini–Hochberg corrections for multiple comparisons when appropriate. Pearson correlation was used to evaluate the strength and direction of association between features.

## 3 Results

### 3.1 Genomic Landscape and Immune Biomarkers

**FIGURE 1:**
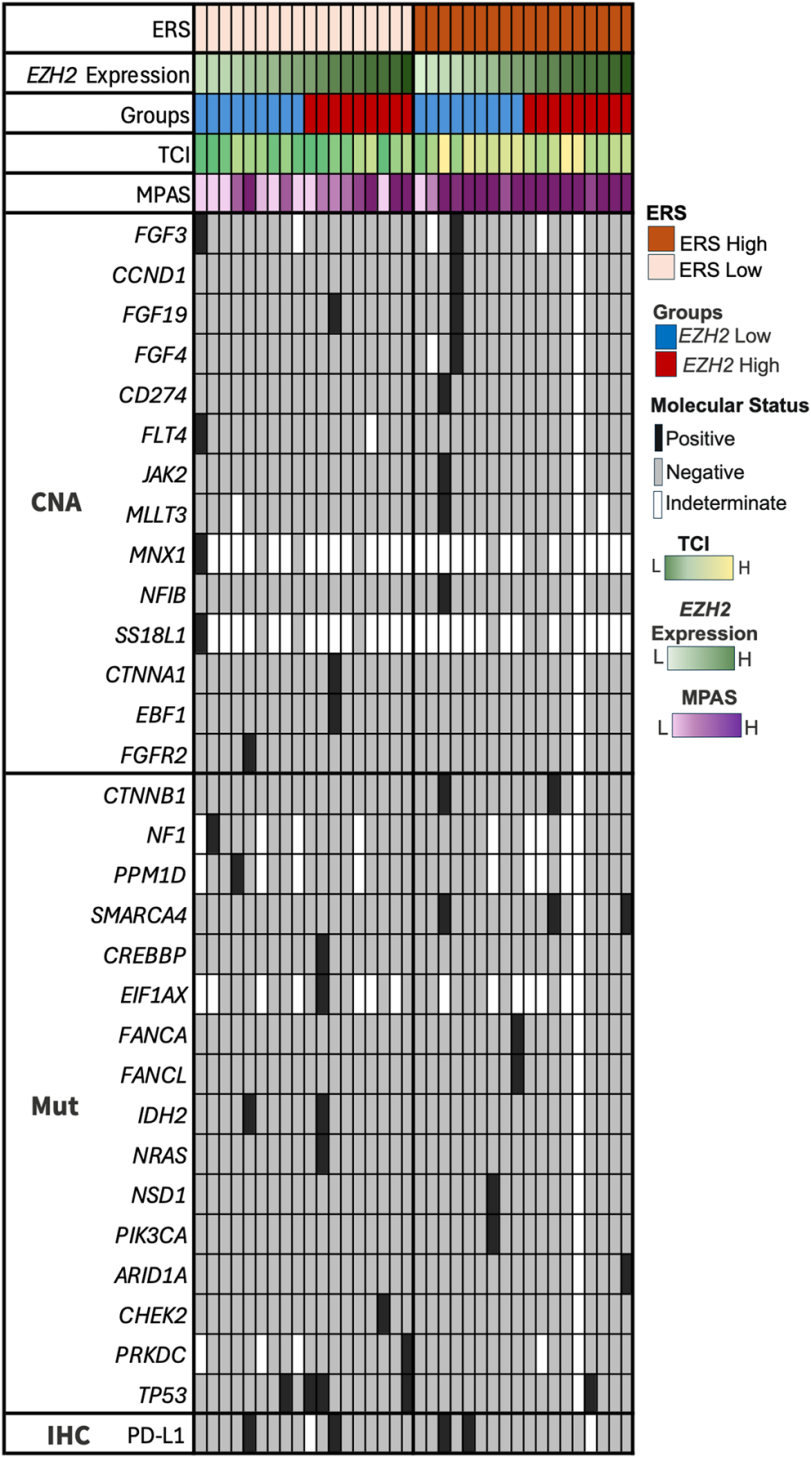
Oncoprint showing prevalence of pathogenic mutations (either point mutation or copy number alteration; black and gray indicate presence and absence of specific alterations, respectively) in olfactory neuroblastoma categorized based on EZH2 repression signature (ERS) into ERS‐low and ERS‐high. Higher T‐cell‐inflamed (TCI) score and higher MAPK activation score (MPAS) were associated with high ERS. CNA, copy number alteration; H, high; IHC, immunohistochemistry; L, low; Mut, mutation. [Color figure can be viewed at wileyonlinelibrary.com]

Four out of 34 (11.7%) ONB samples evaluable for PD‐L1 expression by IHC showed positive staining. No ONB samples were classified as TMB‐high or MSI‐H, in line with previous literature data [2]. Genomic alterations analysis was available for 35 out of 36 cases: only 19 had at least one deleterious genomic alteration (either indels/single nucleotide variants [SNVs] or copy number amplification [CNA]), the most common being a mutation in *TP53* present in 5 out of 35 (14.7%) cases; 2 out of 35 (5.8%) cases harbored mutations in *IDH2* (Figure 1). Median TCI score was −9 (interquartile range, IQR = −86.8 to 38.8), and median MPAS was 0.7 (IQR = −0.3 to 2.2).

### 3.2 EZH2 Mutational Status and Gene Expression in ONB

No pathogenic *EZH2* mutations or CNAs were observed. Median expression of *EZH2* was 44.6 TPM (IQR = 26.13–62.76), significantly higher than the ES cohort, in which median *EZH2* expression was 14.1 TPM (IQR = 8.52–29.47, *p* < 0.0001). EZH2 expression level did not differ between metastatic and primary ONBs (median TPM: 48.4 vs. 43.2, *p* = 0.79) or between neural and basal ONBs (median TPM: 51.9 vs. 42.2, *p* = 0.41).

We stratified ONB samples by the median *EZH2* expression in high (*EZH*‐H) and low (*EZH*‐L) expressing tumors: no genomic alteration had a significantly higher prevalence across EZH2 expression groups. Expression of *MKI67* was significantly higher in *EZH2*‐H tumors (4.07 vs. 3.28 log2TPM + 1, *p* = 0.022); *EZH2* expression did not correlate with the prevalence of PD‐L1 positivity or with gene expression of immune‐checkpoint related biomarkers *PDCD1*/PD‐1 (0.79 vs. 0.59 log2TPM + 1, *p* = 0.93), *CTLA‐4* (0.89 vs. 0.97 log2TPM + 1, *p* = 0.96), and *VSIR*/VISTA (3.86 vs. 3.98 log2TPM + 1, *p* = 0.69), although we observed significantly higher *CD274/*PD‐L1 (2.39 vs. 1.66 log2TPM + 1, *p* = 0.044) gene expression in *EZH2*‐H tumors. There was no significant difference in TCI signature between *EZH2*‐H and *EZH2*‐L tumors, whereas MPAS was higher in *EZH2*‐H tumors (median MPAS: 1.70 vs. 0.33 [AU], *p* = 0.035). With regard to the inferred immune microenvironment, we did not observe any significant differences between *EZH2‐*H and *EZH2*‐L ONB (data not shown).

### 3.3 EZH2 Activity in ONB

**FIGURE 2:**
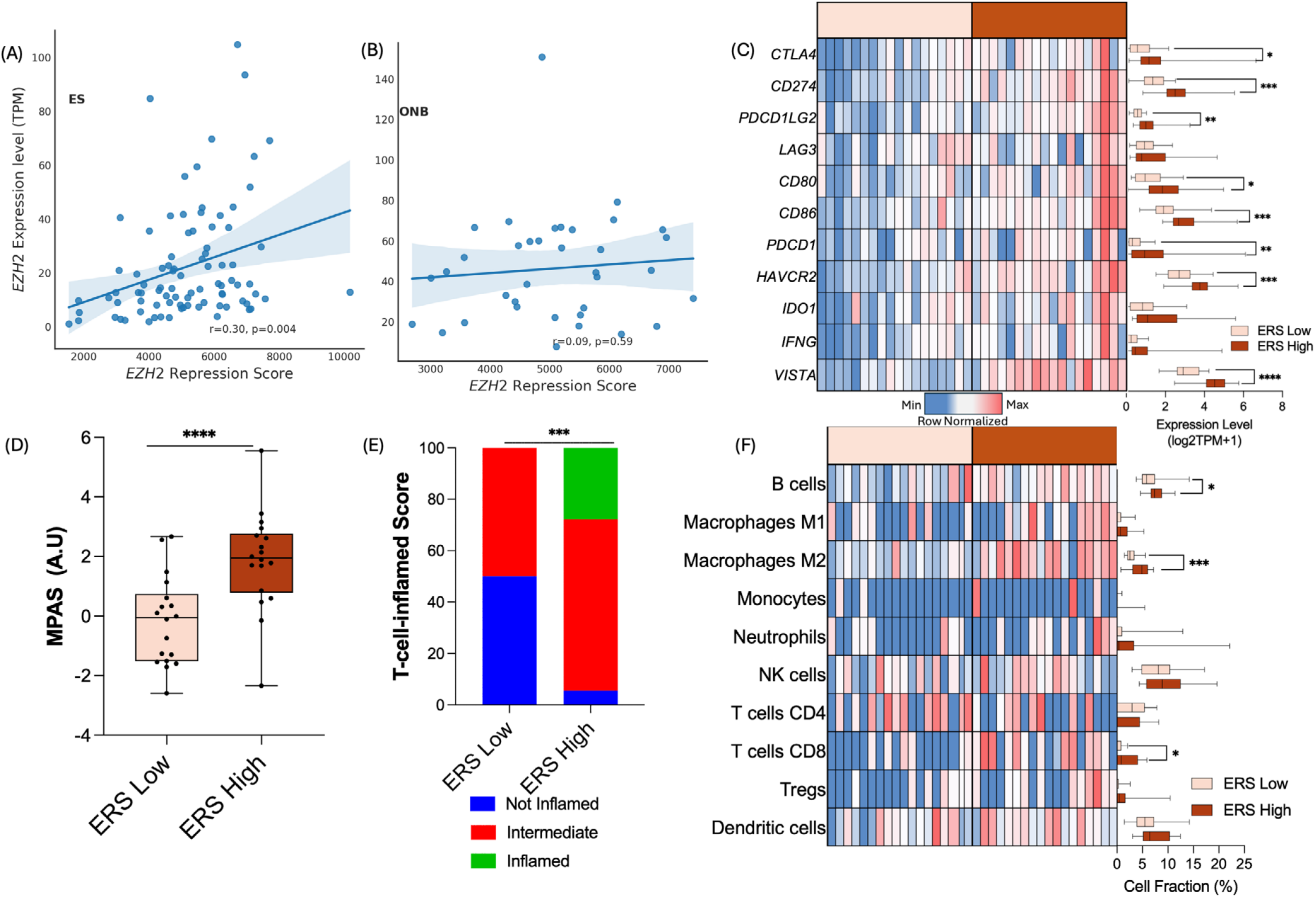
(A) and (B) In both olfactory neuroblastoma (ONB) and epithelioid sarcoma (ES), EZH2 expression did not correlate with EZH2 repression score (ERS). (C) Heatmap showing comparison of immune checkpoint‐related gene expression among ERS‐high and ERS‐low ONB. (D) Higher proportion of T‐cell‐inflamed tumors was seen in ERS‐high ONB compared to ERS‐low ONB (27.8% vs. 0%, *p* < 0.001). (E) ERS‐high tumors displayed a higher MAPK activation score compared with ERS‐low ONB (1.95 vs. −0.06 AU, *p* < 0.001). (F) Heatmap showing inferred microenvironment composition: ERS‐high ONB displayed higher proportion of M2 macrophages, CD8 T cells, and B cells compared with ERS‐low ONB. * = *p* < 0.05, **p < 0.01, ***p < 0.001, and ****p < 0.0001. [Color figure can be viewed at wileyonlinelibrary.com]

To explore EZH2 activity beyond *EZH2* gene expression, we examined the surrogate transcriptional signature ERS (see Supporting Information S1) [17]: low ERS is associated with higher EZH2 methyltransferase activity and high ERS with lower EZH2 methyltransferase activity. ERS score has been previously developed in prostate cancer preclinical models in which inhibition of EZH2 catalytic function resulted in the de‐repression of several genes involved in immune response, T‐cell attraction, and antigen presentation [17]. Median ERS score in ONB was 5099 AU (IQR = 4392–5805), which was similar to ERS in ES (5186 AU [IQR = 4223–6426], *p* = 0.59) and was not correlated with *EZH2* gene expression (Figure 2A,B) in either ONB or ES. ERS score was similar between neural and basal ONB subtypes (Figure S2 ) and was lower in metastatic ONB compared to primary tumors (4542 vs. 5780 AU, *p* = 0.001), potentially suggesting a higher EZH2 methyltransferase activity in metastatic ONB. We explored different variations of the ERS to assess how different weighting strategies affect the scoring approach in the ERS. All ERS variant signatures produced similar results with a strong correlation observed among the scores (Pearson's correlation *r* > 0.85, *p* < 0.0001) (Figure S1A–C). Additionally, the ERS score in our results had a strong correlation with the PRS signature (Pearson's correlation *r* = 0.88, Figure S1D).

We stratified the ONB cohort based on the median ERS score value into high (ERS‐H) and low (ERS‐L) ERS groups, with lower and higher EZH2 activity, respectively. No difference in the proportion of PD‐L1‐positive tumors by IHC was seen between the two groups; four out of five *TP53* mutations were found in ERS‐L ONB (Figure 1). When compared to ERS‐L ONB (Figure 2, Table S2), ERS‐H tumors had increased expression of immune checkpoint genes *CTLA4* (fold change [FC] = 1.71, *p* = 0.03), *PDCD1*/PD‐1 (FC = 3.00, *p* < 0.01), *CD274/* PD‐L1 (FC = 1.79, *p* < 0.01), *TIM‐3* (FC = 1.41, *p* = 0.002), *VSIR*/VISTA (FC = 1.55, *p* < 0.001), and *IRF1* (FC = 1.22, *p* = 0.001).

ERS‐H tumors had increased TCI score (Figure 2D, median 37 vs. −85, *p* < 0.001), with 27.8% “inflamed” tumors vs. 0% in the ERS‐L cohort (*p* = 0.003) and, accordingly, displayed significantly higher transcript levels of HLA Class II genes, Class II‐related trans‐activator gene *CIITA* (median: 3.9 vs. 3.0 log2TPM + 1, *p* < 0.001) and Class I‐related *β2M* (median: 11.0 vs. 9.9 log2TPM + 1, *p* = 0.03). There was a numerically higher median expression of Class I HLA (*HLA‐A*, *HLA‐B*, and *HLA‐C*) in ERS‐H tumors (Table S3). ERS‐H tumors also displayed a higher MPAS (1.95 vs. −0.06 AU, *p* < 0.001; Figure 2E).

Despite overall low immune infiltrates within the tumor microenvironment (Figure 2F, Table S4), ERS‐H was associated with a higher median proportion of M2 macrophage (4.9% vs. 2.5%, *p* < 0.001), B cells (7.3% vs. 5.8%, *p* = 0.047), and CD8^+^ T cells (0.8% vs. 0%, *p* = 0.03), compared with ERS‐L ONB.

**FIGURE 3:**
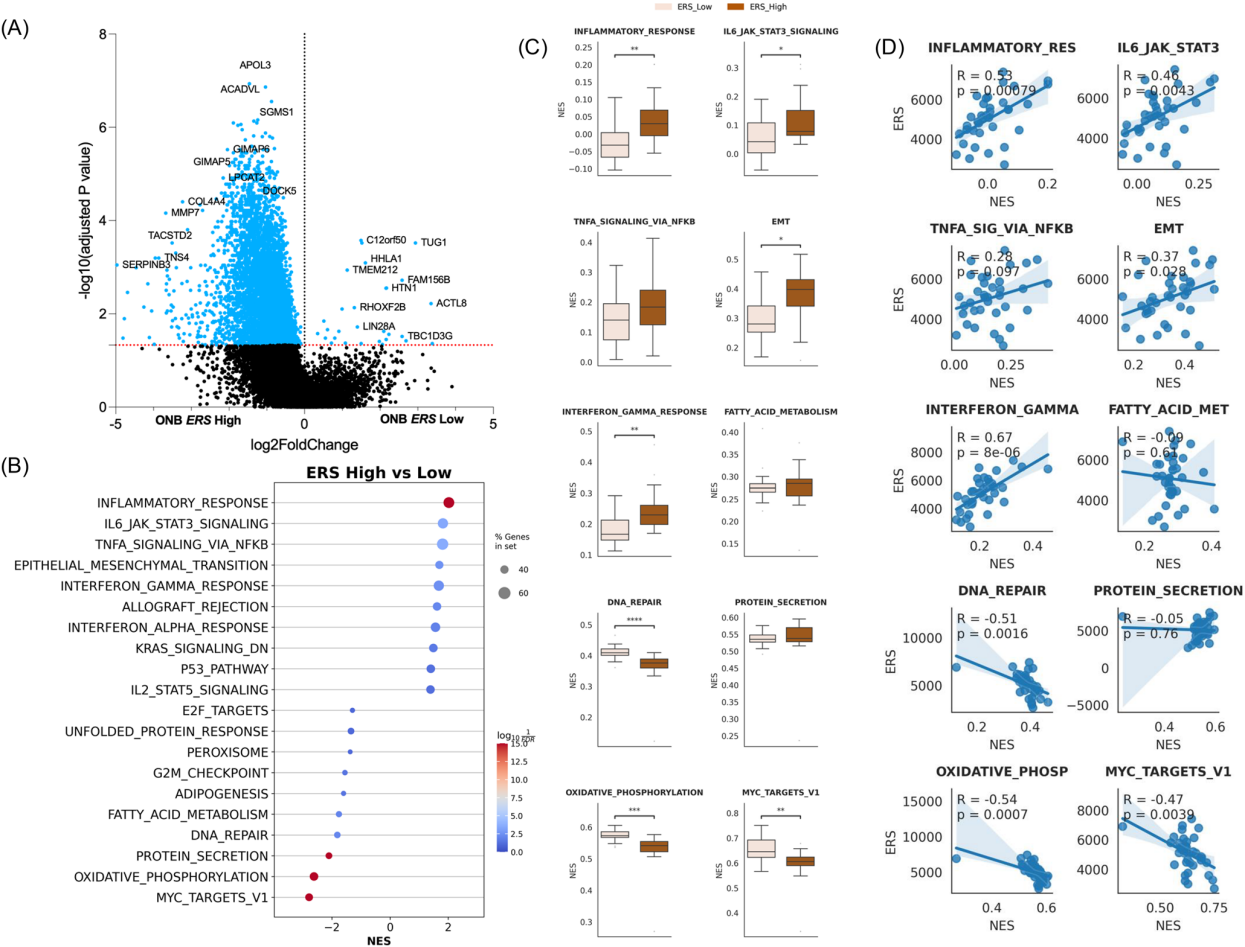
(A) Differential expression analysis between ERS‐low and ERS‐high ONB tumors. (B) Gene set enrichment analysis (GSEA) comparing ERS‐low and ERS‐high ONB. (C) Boxplot showing the distribution of normalized enrichment score (NES) from single sample GSEA (ssGSEA) for the top 10 enriched pathways between ERS‐high and ERS‐low tumors. (D) Correlation between ssGSEA NES of the top 10 pathways and ERS score. [Color figure can be viewed at wileyonlinelibrary.com]

Differential gene expression analysis between ERS‐H and ERS‐L tumors was performed, and the association of ERS with GSEA Hallmark gene sets was examined (Figure 3A–D). We observed significant enrichment of inflammation and immune response‐related hallmark pathways in the ERS‐H cohort, including IL6 JAK STAT3 signaling, interferon alpha response, inflammatory response, interferon gamma response, and TNFA signaling via NFkB (Figure 3B,C). To further assess the strength of this association, we evaluated the correlation between ERS and the normalized enrichment score of the top 10 Hallmark pathways and found a strong positive correlation between ERS and immune‐associated Hallmarks (*R* value range: 0.28–0.67, *p* < 0.05; Figure 3D).

### 3.4 SWI/SNF Pathway in ONB

Given the role of the SWI/SNF complex in regulating EZH2/PRC2 activity [9], we next examined the SWI/SNF‐related genes' mutational status and expression in association with EZH2 repression score. We did not observe any *SMARCB1* mutation in our ONB cohort, whereas three patients displayed pathogenic/likely pathogenic SNV/indel mutations in *SMARCA4* (T910M, K689*, M106fs), with one tumor simultaneously harboring a frameshift pathogenic mutation in *ARID1A* (H1960fs). Of note, two out of three *SMARCA4*‐mutated tumors had *SMARCA4* transcript levels above the overall cohort median, and all cases displayed a high ERS score, suggesting low EZH2 activity. Regarding SWI/SNF‐related gene expression, there was a trend for higher expression of *SMARCB1* in ERS‐H ONB compared to ERS‐L ONB, although not statistically significant, while other SNF/SWI components (*ARID1A*, *ARID1B*, *ARID2*, and *SMARCC2*) had significantly higher expression in ERS‐H ONB.

### 3.5 Validation of ERS in an Independent Cohort

In a validation cohort of 19 ONB samples previously described by Classe et al. [20], the median ERS was 5264 AU (IQR = 4183–5608), similar to our study population. In this cohort, ERS displayed a strong positive correlation with TCI score (Pearson's correlation *r* = 0.81, *p* < 0.001) and a moderate correlation with MPAS score (Pearson's correlation *r* = 0.45, *p* = 0.06). Among 27 patients for whom genomic profiling data were available, deleterious mutations in *ARID1A* and *SMARCA4* were present in four and two cases, respectively, with one patient harboring mutations in both genes. Additionally, one patient harbored a mutation in *SMARCC1*, another component of SWI/SNF.

## 4 Discussion

In a cohort of real‐world ONB patient samples, we observed that low activity of *EZH2*, inferred through a transcriptional repression signature, was able to identify a subset of ONB that showed a higher expression of immune‐related genes, along with higher TCI and MPAS transcriptional signatures, suggesting a potentially greater infiltrate of immune cells and greater susceptibility to immunotherapy and tyrosine kinase inhibitors, respectively. Similar results were observed in an independent ONB dataset, further supporting our findings.

Overexpression of EZH2/PRC2 can promote resistance to immune‐mediated killing in solid and hematologic malignancies, while inactivating *EZH2* mutations have been associated with better response to checkpoint blockade [29]. The mechanism by which EZH2 regulates response to immunotherapy is still unclear: modification of immune checkpoint gene expression, HLA molecules, and IFN‐γ signaling genes expression have all been suggested [12, 13, 14, 30]. Pharmacological EZH2 inhibition has been shown to increase immune cell‐mediated killing and tumor‐infiltrating lymphocytes in several cancer models [13, 16, 17, 31]. In our ONB cohort, low EZH2 activity was associated with increased TCI score, a predictive marker for response to immunotherapy (pembrolizumab), increased HLA Class II and β2m expression, and a trend toward increased HLA Class I expression. These findings suggest that low EZH2 activity status may identify a subgroup of ONB, a tumor known to be immunologically cold, with increased tumor inflammation and, potentially, susceptibility to checkpoint blockade or other immunotherapies. Of note, we observed an upregulation of immune‐related pathways in ERS‐H tumors, including “IL6/JAK/STAT3 signaling,” “IFNγ response,” and “IFNα response” pathways, which correlate with enhanced T cell activation [32, 33]. Findings regarding the TCI score were similar in a publicly available independent validation dataset by Classe et al. [20]. Of note, the epithelial‐to‐mesenchymal transition (EMT) signature, which is known for its association with tumor aggressiveness and immunologically exhausted microenvironment, was also enriched in ERS‐H ONB tumors, in line with studies suggesting that inhibition of *EZH2* promoted enrichment in EMT pathways [34, 35].

In our study, the estimated proportion of M2 macrophages and, to a lesser extent, B cells and CD8^+^ T cells was increased in the tumor microenvironment of ONB with low EZH2 activity. The impact of EZH2 inhibition on tumor‐associated macrophages polarization is unclear: according to Wang et al. [36], administration of tazemetostat or GSK126, another EZH2‐specific enzymatic inhibitor, was associated with an increase in polarization and infiltration of M2 macrophages in both in vitro and in vivo models of breast cancer, whereas *EZH2* knockdown exerted the opposite effect. This difference was suggested to be explained by the indirect, noncanonical activity of EZH2 in regulating M2‐promoting chemokines like CCL2, which is not directly affected by EZH2 methyltransferase inhibition but is lost upon *EZH2* gene knockdown. Nevertheless, an increase in immunosuppressive M2 macrophages in the tumor‐associated microenvironment is of interest as it might promote an immunosuppressive environment and potentially counterbalance immunotherapy efficacy.

MAPK signaling is crucial for cancer proliferation and survival. The MPAS is a transcriptional signature shown to predict sensitivity to MAPK inhibitors in in vitro models, validated in retrospective clinical data analysis [22]. Importantly, MPAS does not correlate with RAS/RAF mutational status, thus might identify subgroups of patients who would benefit from MAPK inhibitors regardless of mutational profile. Riquelme et al. showed that different amino acid substitutions in mutant KRAS modulate EZH2 expression in different ways, either through the MEK–ERK signaling pathway or through the PI3K/AKT signaling pathway. They also observed that EZH2 inhibition increased the sensitivity to MEK–ERKi and PI3K/AKTi based on specific KRAS mutations, overall suggesting that EZH2 is a downstream effector of KRAS signaling in malignant cells harboring *KRAS* mutations [37]. In our study, high *EZH2* expression and low EZH2 activity also corresponded to increased MPAS.

The mechanism of regulation of EZH2 activity in ONB is unknown to date. In the tested cohorts, none of the samples harbor any *EZH2* or *SMARCB1* mutations; nevertheless, ERS in ONB was comparable to ES, a *SMARCB1*‐deficient cancer in which the EZH2 pathway is known to be dysregulated due to the loss of *SMARCB1* at either the genomic or protein level [10]. A lower gene expression of other SWI/SNF components, including *ARID1A, ARID1B, ARID2*, and *SMARCC2*, was seen in ONB with high *EZH2* activity (ERS‐L), and mutations in other SWI/SNF components, including *SMARCA4* and *ARID1A*, were seen in a minority of patients, in line with previous literature [38, 39], although their biological significance is still undetermined.

Several EZH2 inhibitors are currently under study; tazemetostat, a methyltransferase inhibitor, is the only FDA‐approved drug to date, and its current indications include ES, where it yields 15% of objective response as monotherapy [10]. The relatively low response rate in ES suggests the existence of both compensatory mechanisms [40] and a potential role of nonenzymatic activity of EZH2, not affected by tazemetostat, in promoting disease progression: EZH2 degraders such as proteolysis targeting chimeras, which would target both the noncanonical and the methyltransferase activity of EZH2, might potentially overcome the current limitations [41]. Additionally, preclinical studies focusing on promoting the stabilization of the SWI/SNF complex rather than directly targeting EZH2 are being evaluated [42]. EZH2 inhibitors are currently under study in combination with pembrolizumab in urothelial carcinoma (NCT03854474), aggressive B‐cell lymphoma (NCT06242834), and non‐small cell lung cancer (NCT05467748, NCT06644768).

Limitations of our study include the unavailability of detailed clinical annotation, which prevents correlation with tumor grading and treatment outcomes. In addition, the EZH2 repression signature used in this study was established in prostate cancer using indirect EZH2 inhibition instead of EZH2‐specific enzymatic inhibitors such as tazemetostat. Finally, and most importantly, our analysis of the *EZH2* expression, ERS, and immune infiltrate is based on deconvolution of bulk RNA‐seq, without data on spatial resolution, and no IHC staining of the EZH2 protein was available to validate the correlation with ERS and immune‐related findings.

In conclusion, based on this preliminary observation, ONB tumors with low EZH2 repression signature and high EZH2 activity may be susceptible to treatment with EZH2 inhibitors, which might in turn increase susceptibility to immunotherapy; further in vitro and functional studies, including IHC validation, are needed to validate these findings preclinically and translate to a potential therapeutic approach for ONB.

## Author Contributions

E.X. and C.S.F. designed the study and interpreted the results. T.A. and H.K. collected data and performed formal analysis. T.A. prepared the figures, E.X. wrote the first version of the manuscript. C.S.F. provided scientific expertise, reviewed the paper, and supervised the study. T.A., H.K., A.E., R.M., H.S., E.L., A.V., D.S., D.B.‐C., N.R.L., J.L.G. reviewed and edited the paper. All authors approved the final version of the manuscript.

## Ethics Statement

Ethical review and approval were waived for this study. Per NIH Office of Human Subjects Research Protections (OHSRP) Policy memo (1/15/2019) regarding the NIH Intramural Research Program (IRP) of the 2019 Common Rule (45 CFR 46, Sub‐part A), for all NIH research initiated on or after January 21, 2019, formal determination by OHSRP that NIH IRP research is not human subjects research is not mandatory for research activities involving only de‐identified (not individually identifiable) human specimens or data.

## Consent

The authors have nothing to report.

## Conflicts of Interest

T.A., A.E., A.V., and D.S. reported employment from Caris. N.R.L. Jr. received research funding from Merck not related to this study. The other authors declare no conflicts of interest.

## Supporting information


**Table S1:** 29‐gene list used to calculate EZH2 repression score, as described by Morel et al. [1].
**Table S2:** Comparison of immune‐related gene expression expressed in TPM (log2 + 1) between ERS‐low and ERS‐high ONB.
**Table S3:** Comparison of HLA expression expressed in TPM (log2 + 1) between ERS‐low and ERS‐high ONB.
**Table S4:** Estimated proportion (quanTIseq) of Immune Infiltrate in ONB stratified by ERS.
**Figure S1:** Correlation between different EZH2 repression scores.
**Figure S2:** (A) ONB samples divided into neural and basal subtypes per Classe et al. [3], using Ward's clustering method as described previously [4]. (B) No difference in ERS was seen between neural and basal subtypes.

## Data Availability

Data will be made available upon reasonable request with the permission of Caris Life Science. Individual participant data are not available for sharing.
