# Supplementary material for: Genomic and Immune Correlates of EZH2 Expression and Activity in Olfactory Neuroblastoma
Source: Head Neck. 2025 Nov 12;48(4):932–40. doi: 10.1002/hed.70076 (PMC12972649; doi:10.1002/hed.70076)
Supplement: Supplementary file 1 — Table S1: 29‐gene list used to calculate EZH2 repression score, as described by Morel et al. [1]. Table S2: Comparison of immune‐related gene expression expressed in TPM (log2 + 1) between ERS‐low and ERS‐high ONB. Table S3: Comparison of HLA expression expressed in TPM (log2 + 1) between ERS‐low and ERS‐high ONB. Table S4: Estimated proportion (quanTIseq) of Immune Infiltrate in ONB stratified by ERS. Figure S1: Correlation between different EZH2 repression scores. Figure S2: (A) ONB samples divided into neural and basal subtypes per Classe et al. [3], using Ward's clustering method as described previously [4]. (B) No difference in ERS was seen between neural and basal subtypes. [file HED-48-932-s001.docx]

**Supplementary Materials**

**Table S1 –** 29-gene list used to calculate EZH2 repression score, as described by Morel et al. [1]

| **Gene** | **Weights** |
| --- | --- |
| **IFIT1** | 189.5737206 |
| **MCAM** | 126.9552146 |
| **LGALS3BP** | 104.5763895 |
| **XAF1** | 103.2764477 |
| **IRF7** | 102.3259138 |
| **CXCL10** | 94.91667878 |
| **PPBP** | 93.07112204 |
| **ISG15** | 87.77826856 |
| **HR** | -81.50172117 |
| **KRT10** | 69.51001869 |
| **BST2** | 56.71217579 |
| **RSAD2** | 55.69539853 |
| **IFI44** | 51.66033176 |
| **OAS3** | 43.00782536 |
| **KRT1** | 41.06430867 |
| **LGALS9** | 39.61557658 |
| **DHX58** | 36.77590278 |
| **RTP4** | 36.66686165 |
| **SCGB1A1** | 36.45179926 |
| **MX2** | 35.51494032 |
| **GBP7** | 32.48968587 |
| **DDX60** | 30.35789776 |
| **IFIT3** | 26.45578194 |
| **SP100** | 25.93567214 |
| **CMPK2** | 22.59376575 |
| **GBP3** | 21.46126774 |
| **OAS2** | 17.90711659 |
| **ZBP1** | 16.06445844 |
| **NLRC5** | 15.55581122 |

**Table S2 –** Comparison of immune-related gene expression expressed in TPM (log2 +1) between ERS-low and ERS-high ONB

| **IO-Related Genes** | **Median (ERS Low)** | **Median (ERS High)** | **p-value** | **q-value** |
| --- | --- | --- | --- | --- |
| *PDCD1LG2* | 0.57 | 0.99 | 0.0013 | 0.0029 |
| *IFNG* | 0.28 | 0.50 | 0.0604 | 0.0739 |
| *CD274* | 1.35 | 2.50 | 0.0002 | 0.0008 |
| *CTLA4* | 0.73 | 1.15 | 0.0238 | 0.0327 |
| *LAG3* | 0.92 | 0.78 | 0.8124 | 0.8124 |
| *C10orf54* | 2.91 | 4.52 | 0.0000 | 0.0000 |
| *CD86* | 1.91 | 2.68 | 0.0007 | 0.0018 |
| *CD80* | 0.96 | 1.85 | 0.0155 | 0.0244 |
| *HAVCR2* | 2.71 | 3.76 | 0.0005 | 0.0018 |
| *PDCD1* | 0.47 | 1.53 | 0.0030 | 0.0056 |
| *IDO1* | 0.88 | 1.09 | 0.1334 | 0.1467 |

**Table S3-** Comparison of HLA expression expressed in TPM (log2 +1) between ERS-low and ERS-high ONB

| ***HLA - Genes*** | **Median**  **(ERS Low)** | **Median**  **(ERS High)** | **p-value** | **q-value** |
| --- | --- | --- | --- | --- |
| *HLA-C* | 7.20 | 7.54 | 0.050 | 0.120 |
| *HLA-DQB3* | 0.41 | 0.46 | 0.400 | 0.480 |
| *HLA-DPA3* | 1.79 | 2.55 | 0.129 | 0.193 |
| *HLA-DRA* | 6.46 | 7.76 | 0.000 | 0.001 |
| *HLA-DQB1* | 3.86 | 4.72 | 0.004 | 0.015 |
| *HLA-DQA1* | 3.78 | 4.91 | 0.003 | 0.015 |
| *HLA-DPB2* | 0.32 | 0.42 | 0.603 | 0.603 |
| *HLA-B* | 6.97 | 7.47 | 0.092 | 0.175 |
| *HLA-DQA2* | 0.95 | 1.56 | 0.185 | 0.247 |
| *HLA-DQB1-AS1* | 0.29 | 0.20 | 0.556 | 0.603 |
| *HLA-A* | 7.41 | 7.59 | 0.029 | 0.088 |
| *HLA-DQB2* | 0.74 | 1.25 | 0.102 | 0.175 |
| *HLA-DRB9* | 0.40 | 0.48 | 0.857 | 0.897 |
| *HLA-DRB6* | 0.22 | 0.13 | 0.792 | 0.897 |
| *HLA-DRB7* | 0.20 | 0.50 | 0.485 | 0.762 |
| *HLA-DRB2* | 0.15 | 0.29 | 0.193 | 0.353 |
| *HLA-DRB8* | 0.48 | 0.64 | 0.762 | 0.897 |
| *HLA-DPA1* | 6.27 | 7.58 | 0.000 | 0.000 |
| *CIITA* | 3.00 | 3.94 | 0.000 | 0.000 |
| *HLA-DRB3* | 2.27 | 1.25 | 0.897 | 0.897 |
| *HLA-DRB1* | 5.29 | 6.48 | 0.000 | 0.000 |
| *HLA-DPB1* | 5.26 | 6.79 | 0.000 | 0.000 |
| *HLA-DRB5* | 0.67 | 1.77 | 0.088 | 0.194 |

**Table S4 -** Estimated proportion (quanTIseq) of Immune Infiltrate in ONB stratified by ERS

| **quanTIseq cell fractions** | **Median (ERS Low)** | **Median (ERS High)** | **p-value** | **q-value** |
| --- | --- | --- | --- | --- |
| Monocytes | 0.00% | 0.00% | 0.545 | 0.545 |
| B cells | 5.79% | 7.34% | 0.014 | 0.047 |
| Neutrophils | 0.00% | 0.35% | 0.331 | 0.369 |
| Macrophages M2 | 2.53% | 4.89% | 0.000 | 0.004 |
| NK cells | 8.11% | 8.90% | 0.333 | 0.369 |
| Dendritic cells | 5.49% | 6.43% | 0.168 | 0.257 |
| T cells CD4 | 2.97% | 0.00% | 0.180 | 0.257 |
| Tregs | 0.00% | 0.45% | 0.091 | 0.195 |
| Macrophages M1 | 0.00% | 0.67% | 0.098 | 0.195 |
| T cells CD8 | 0.00% | 0.84% | 0.007 | 0.035 |

**Figure S1 –** Correlation between different EZH2 repression scores.

Correlation is shown between ERS and A) equally weighted ERS, for which all genes were assigned a uniform weight of 1, B) ERS calculated by excluding immune regulatory genes (IFIT1, MCAM, LGALS3BP, XAF1, IRF7, CXCL10, ISG15, IFI44, OAS3, RTP4, SCGB1A1, MX2, GBP7, DDX60, IFIT3, SP100, OAS) and C) both. Very strong positive correlation was in all three cases. D) ERS score appeared to have a strong positive correlation with polycomb repression signature reported by Yu et al [2].


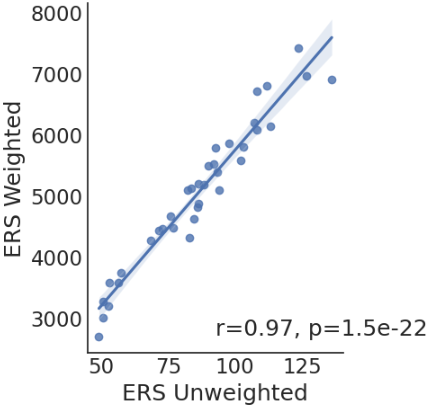

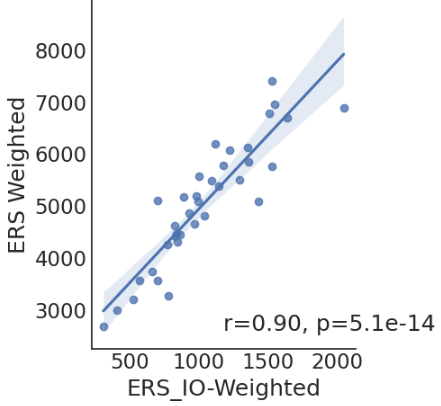

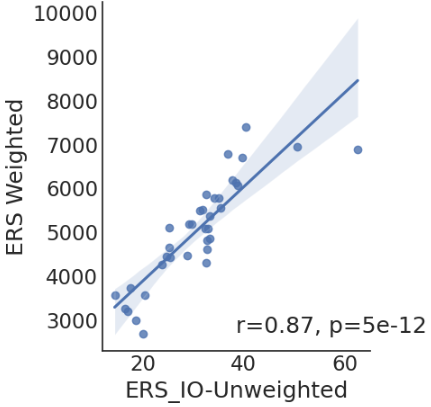

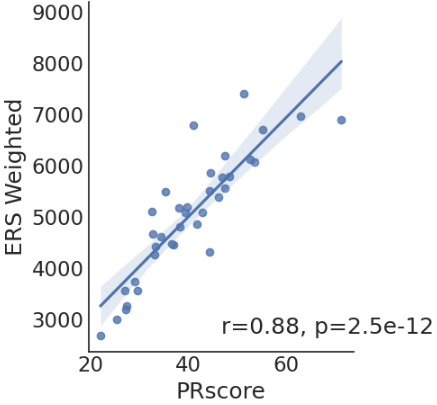


**A**

**D**

**B**

**Figure S2 -** A) ONB samples divided into neural and basal subtypes per Classe et al[3]., using Ward’s clustering method as described previously [4] B) No difference in ERS was seen between neural and basal subtypes


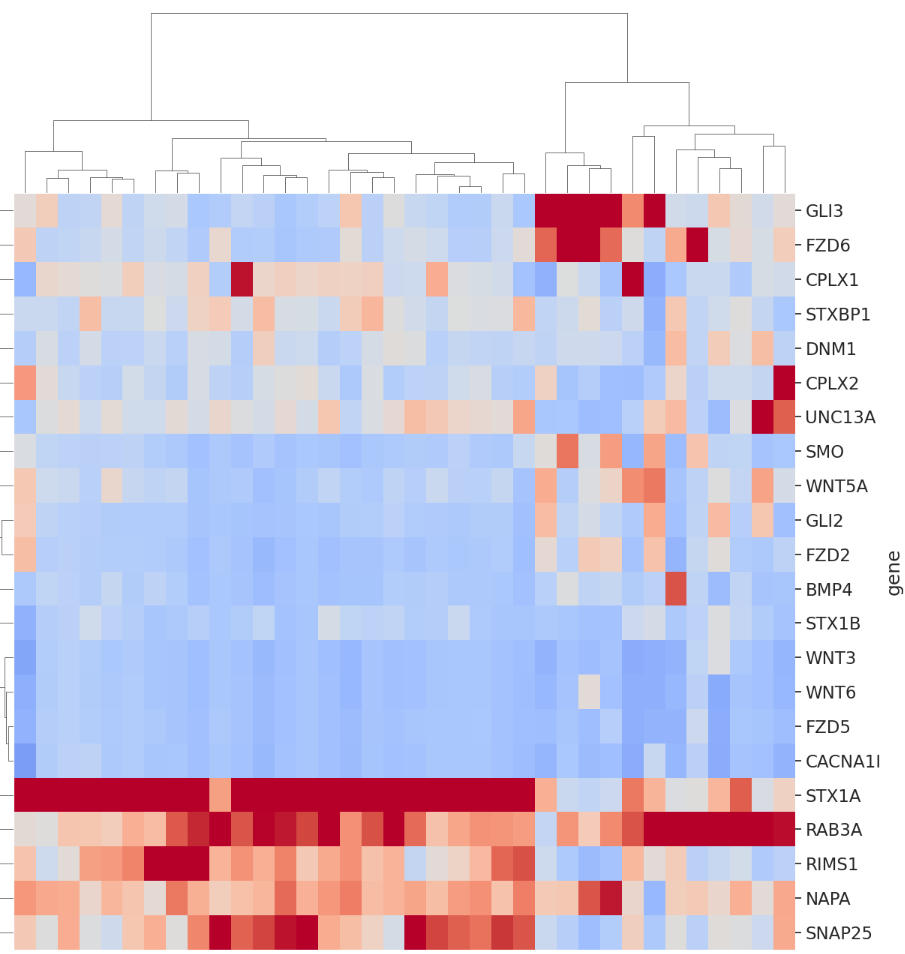


Basal

Neural

**A**

**B**

**References**

1. Morel KL, Sheahan AV, Burkhart DL, Baca SC, Boufaied N, Liu Y, et al. EZH2 inhibition activates a dsRNA-STING-interferon stress axis that potentiates response to PD-1 checkpoint blockade in prostate cancer. Nat Cancer. 2021 Apr;2(4):444-56. PMID: 33899001. doi: 10.1038/s43018-021-00185-w.

2. Yu J, Yu J, Rhodes DR, Tomlins SA, Cao X, Chen G, et al. A polycomb repression signature in metastatic prostate cancer predicts cancer outcome. Cancer Res. 2007 Nov 15;67(22):10657-63. PMID: 18006806. doi: 10.1158/0008-5472.CAN-07-2498.

3. Classe M, Yao H, Mouawad R, Creighton CJ, Burgess A, Allanic F, et al. Integrated Multi-omic Analysis of Esthesioneuroblastomas Identifies Two Subgroups Linked to Cell Ontogeny. Cell Rep. 2018 Oct 16;25(3):811-21 e5. PMID: 30332658. doi: 10.1016/j.celrep.2018.09.047.

4. Xue E, Bracken-Clarke D, Krause H, Adeyelu T, Evans MG, Akbulut D, et al. Characterization of Somatostatin Receptor 2 Gene Expression and Immune Landscape in Sinonasal Malignancies. Cancers (Basel). 2024 Nov 24;16(23). PMID: 39682120. doi: 10.3390/cancers16233931.
